# Mapping Metabolic Brain Activity in Three Models of Hepatic Encephalopathy

**DOI:** 10.1155/2013/390872

**Published:** 2013-03-14

**Authors:** Natalia Arias, Marta Méndez, Camino Fidalgo, María Ángeles Aller, Jaime Arias, Jorge L. Arias

**Affiliations:** ^1^Laboratorio de Neurociencias, Departamento de Psicología, Universidad de Oviedo, Plaza Feijoo s/n., 33003 Oviedo, Spain; ^2^Departamento de Cirugía I, Facultad de Medicina, Universidad Complutense de Madrid, Ciudad Universitaria s/n., 28040 Madrid, Spain

## Abstract

Cirrhosis is a common disease in Western countries. Liver failure, hyperammonemia, and portal hypertension are the main factors that contribute to human cirrhosis that frequently leads to a neuropsychiatric disorder known as hepatic encephalopathy (HE). In this study, we examined the differential contribution of these leading factors to the oxidative metabolism of diverse brain limbic system regions frequently involved in memory process by histochemical labelling of cytochrome oxidase (COx). We have analyzed cortical structures such as the infralimbic and prelimbic cotices, subcortical structures such as hippocampus and ventral striatum, at thalamic level like the anterodorsal, anteroventral, and mediodorsal thalamus, and, finally, the hypothalamus, where the mammillary nuclei (medial and lateral) were measured. The severest alteration is found in the model that mimics intoxication by ammonia, followed by the thioacetamide-treated group and the portal hypertension group. No changes were found at the mammillary bodies for any of the experimental groups.

## 1. Introduction

Portal hypertension is one of the main complications of human cirrhosis that frequently leads to a neuropsychiatric disorder known as hepatic encephalopathy (HE). The genesis of portal hypertension implies an increase in vascular resistance that can occur at any level within the portal venous system [1]. Elevated blood flow and brain ammonia levels have been strongly implicated in the pathogenesis of HE [2]. Ammonia is a common etiological factor in HE as well as in various hyperammonemic conditions, including inborn errors of the urea cycle, Reye's syndrome, valproate toxicity, idiopathic hyperammonemia, and other conditions [3–6]. Elevated ammonia and its chief metabolite, glutamine, are believed to be important factors responsible for altered cerebral functions, including multiple neurotransmitter system failures, altered bioenergetics, and oxidative stress [7].

Likewise, patients with liver disease have HE, which incorporates a spectrum of manifestations including psychomotor dysfunction, increased reaction time, sensory abnormalities, and poor concentration [8]. Depending on the definition used, HE prevalence varies between 30–84% in patients with cirrhosis [9], a common disease in Western countries [10]. In humans, few studies have been carried out on memory alterations in patients with cirrhosis who develop HE, and, although some authors argue that memory disturbances are not a major symptom of HE [11], others state that patients with HE present clear mnesic alterations. Hence, Bahceci et al. [12] found a poorer performance in several memory tests in patients with cirrhosis, whereas Ortiz et al. [13] showed a learning deficit and impairment in long-term memory. 

Taken together, liver disease, portal hypertension, and hyperammonemia appear to be the main contributing factors that lead to the occurrence of HE.

According to the proposal of Butterworth et al. [14], HE is reclassified into different types, depending on its origin or cause. Type B concerns HE related to portosystemic shunt, which does not necessarily involve any hepatocytic alteration and includes portocaval shunt, partial ligature of the portal vein, and triple portal vein ligation [15]. Type C is associated with chronic liver failure (cirrhosis). As HE can be derived from different causes, diverse experimental models have been developed to reproduce the characteristics of HE as closely as possible and to study brain dysfunction in this syndrome [14]. In this study, we try to isolate the main factors leading to HE through the use of three experimental models: portal hypertension, hyperammonemia, and thioacetamide. Portal hypertension by triple portal vein ligation [16] involves collateral circulation but with a discrete hepatocellular insufficiency. The model of hyperammonemia [17] allowed us to study the effect of ammonia as a toxic brain substance but it lacks also liver failure [18]. Therefore, to explore cirrhosis, a model of chronic thioacetamide administration was used [19].

Difficulties learning diverse types of tasks have been shown in these experimental models, such as classical conditioning [20], conditional discrimination [21, 22], reference [23], and working memory [24]. However, less is known about the specific role of liver failure, portal hypertension, and hyperammonemia in the development of bioenergetic brain disturbances.

The aim of this study is to elucidate the differential contribution of these leading factors to the oxidative metabolism of diverse brain limbic system regions frequently involved in memory process by histochemical labelling of cytochrome oxidase (COx). COx is a mitochondrial enzyme involved in the phosphorylation process that generates ATP. This energy is used to maintain the resting membrane potential and the synthesis of molecules and neurotransmitters, among other functions [25]. Because metabolic activity is tightly coupled with neuronal activity, this technique can be used as an index of regional functional activity in the brain, reflecting changes in tissue metabolic capacity induced by sustained energy requirements of the nervous system associated with learning [23, 26].

## 2. Material and Methods

### 2.1. Subject

A total of 28 male adult Wistar rats were used (230–260 g at the start of the experiments) from the animalarium of Oviedo University. All the animals had ad libitum access to food and tap water and were maintained under standard laboratory conditions (20–22°C, 65–70% relative humidity, and a 12 h light/dark cycle). The procedures and manipulation of the animals used in this study were carried out according to the European Parliament and the Council of the European Union 2010/63/UE and were approved by the Oviedo University committee for animal studies.

### 2.2. Procurement of Experimental Models

The animals were randomly distributed into 3 groups: portal hypertension (PH group, *n* = 12), hyperammonemia (HA group, *n* = 8), and animals with cirrhosis by administration of thioacetamide (TAA group, *n* = 8). The surgical procedures and protocols used for the different experimental models are described below.

#### 2.2.1. Portal Hypertension

The portal hypertension model was carried out under induction of anaesthesia by i.m. injection of ketamine (100 mg/kg) and xylacine (12 mg/kg). A midline abdominal incision was performed and a part of the intestinal loops was gently shifted to the left and covered with saline-moistened gauze. The portal vein was isolated along its length. Portal hypertension was produced by triple partial ligation [16]. Three stenosing ligatures were performed in the superior, medial, and inferior portions of the portal vein, respectively, and maintained in position by the previous fixation of the ligatures to a sylastic guide. The stenoses were also calibrated by a simultaneous ligature (3–0 silk) around the portal vein and a 20 G needle. The abdominal incision was closed in two layers with an absorbable suture (polyglycolic acid) and 3–0 silk. With respect to postsurgical care, the rats were maintained close to a source of heat until they recovered consciousness (10–15 min) to avoid postoperative hypothermia. Brains were assessed 45 days later.

#### 2.2.2. Thioacetamide

The method used to produce cirrhosis was weekly administration of thioacetamide (Sigma, Germany) in drinking water as described by Li et al. [19]. The thioacetamide (TAA) was administered for 12 weeks and its concentration was modified weekly depending on the animals' weight gain or weight loss. The initial concentration of TAA was 0.03%. After twelve weeks of treatment, they were placed in groups of 5 rats per cage and they were assessed after two weeks. During this period, the animals received TAA at 0.04%.

#### 2.2.3. Hyperammonemia

The method used to produce hyperammonemia was performed according to Azorín et al. [17]. Briefly, the animals were fed with a standard diet supplemented with ammonium acetate ad libitum for up to 27 days.

### 2.3. Cytochrome Oxidase Histochemistry

90 minutes after the performance of a spatial reference memory task, animals were decapitated, and the brains were removed intact, frozen rapidly in isopentane (Sigma-Aldrich), and stored at −40°C. Coronal sections (30 *μ*m) of the brain were cut at −20°C in a cryostat (Leica CM1900, Germany) and were mounted on slides.

We used a quantitative COx histochemistry described by Gonzalez-Lima and Cada [25]. To quantify enzymatic activity and to control staining variability across different baths of staining, sets of tissue homogenate standards obtained from Wistar rat brains were included with each bath of slides. These standards were cut at different thicknesses (10, 30, 40, and 60 *μ*m).

Sections and standards were incubated for 5 min in 0.1 M phosphate buffer with 10% w/v sucrose and 0.5% v/v glutaraldehyde, pH 7.6. After this, four baths of 0.1 M phosphate buffer with 10% w/v sucrose were given for 5 min each. Then 0.05 M Tris buffer, pH 7.6, with 275 mg/L cobalt chloride, 10% w/v sucrose, and 0.5% v/v dimethylsulfoxide were applied for 10 min, to enhance staining contrast. Subsequently, sections and standards were incubated in a solution of 0.06 g cytochrome c (Sigma, St. Louis, MO, USA), 0.016 g catalase, 40 g sucrose, 2 mL dimethylsulfoxide, and 0.4 g diaminobenzidine tetrahydrochloride in 800 mL of 0.1 M phosphate buffer, at 37°C for 1 h. The reaction was stopped by fixing the tissue in buffered formalin for 30 min at room temperature with 10% w/v sucrose and 4% v/v formalin. Finally, the slides were dehydrated in series of ethanol baths (from 30% to 100% v/v ethanol), cleared with xylene, and coverslipped with Entellan (Merck, Germany).

#### 2.3.1. Quantification

Quantification of COx histochemical staining intensity was done by densitometric analysis using a computer-controlled image analysis workstation (MCID, InterFocus Imaging Ltd., Linton, England) made up of a high-precision illuminator, a digital camera, and a computer with specific software for image analysis. The mean optical density (OD) of each structure was measured on the right side of the bilateral structures using three consecutive sections of each animal. In each section, four nonoverlapping readings were taken using a square-shaped sampling window that was adjusted for each region size. A total of twelve measurements were taken per region. These twelve measurements were averaged to obtain one mean per region for each subject. OD values were then converted to COx activity units, determined by the enzymatic activity of the standards which were measured spectrophotometrically [25]. Measurements were performed by an investigator blind to the groups.

**Figure 1:**
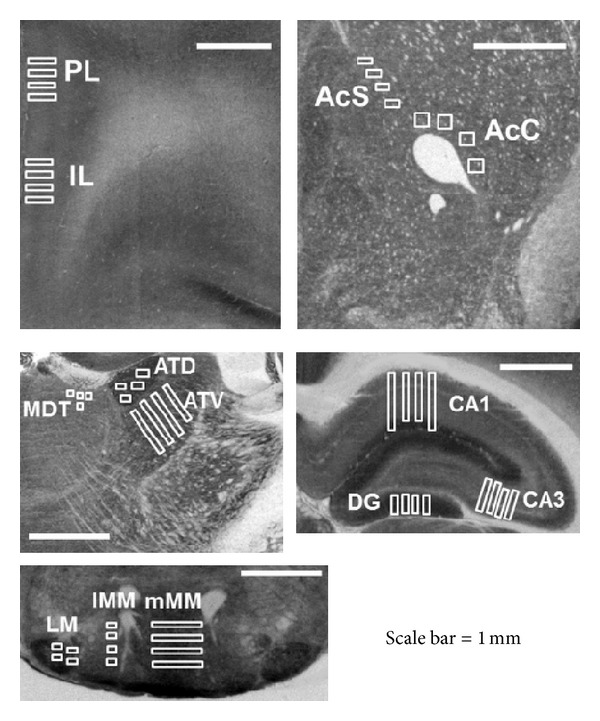
Cytochrome oxidase (COx) histochemistry in the sampled regions in which the squares used to take the measures were presented. Prelimbic (PL) and infralimbic (IL) cortex, accumbens core and shell (AcC and AcS), thalamic nuclei (ADT, AVT, and MDT), dorsal hippocampus (CA1, CA3, and DG) and Mammillary nuclei (mMM, lMM, and LM). Scale bar: 1 mm.

The regions of interest were anatomically defined according to Paxinos and Watson (2005) [27]. The regions of interest and the distance in mm of the regions counted from bregma were: +3.20 mm for prefrontal cortex (the infralimbic cortex and prelimbic cortex (IL and PL, resp.), +2.28 mm for ventral striatum (accumbens core and shell, AcC and AcS), −1.40 for the anterodorsal thalamus (ADT), the anteroventral thalamus (AVT), and the mediodorsal (MDT), −1.20 mm for the CA1, CA3, and the dentate gyrus (DG) subfields of the dorsal hippocampus, and +4.52 mm for the medial part of the medial mammillary nucleus (mMM), the lateral part of the medial mammillary nucleus (lMM), and the lateral mammillary nucleus (LM). See Figure 1.

### 2.4. Data Analysis

All data were analyzed in the Sigma-Stat 3.2 program (Systat, Richmond, USA) and were expressed as mean ± SEM. A one-way analysis of variance was performed (factor: group). When a significant effect was found post hoc paired Tukey's test was carried out. The results were considered as statistically significant if *P* < 0.05.

## 3. Results

The post hoc paired Tukey's tests showed differences between groups in their prefrontal cortex COx activity. The HA group showed higher activity than the PH and TAA groups in the PL (F2, 24 = 47.674) (*P* < 0.001), and the TAA group also showed higher activity than the PH group (*P* < 0.001). In the IL, the same difference between the HA group and the other groups was found (F2, 24 = 53.649, *P* < 0.001). In the ventral striatum, the same pattern of metabolic activity was found in the AcC and AcS, with the HA group showing higher activity, followed by the TAA group, which differed from the PH group (F2, 25 = 69.662, *P* < 0.001; F2, 25 = 76.819, *P* < 0.01, resp.). These differences were also found in the ADT (F2, 25 = 63.441, *P* < 0.001). However, in the AVT (F2, 25 = 13.610, *P* < 0.05) and MDT (F2, 25 = 27.970, *P* < 0.001), differences were only found when comparing the HA group with the other experimental groups, with the former group being the most highly activated. Examining the dorsal hippocampus, in the DG, the HA group showed higher activity than the PH and TAA groups (F2, 25 = 47.402, *P* < 0.01), and the TAA group showed greater activity than the PH group (*P* < 0.001). Similarly, in CA3, the HA group was the most highly activated in relation to the other two groups (F2, 25 = 63.059, *P* < 0.001), and the TAA group showed lower activity than the PH group (*P* < 0.001). In CA1, the highest metabolic activity was found in the HA group, which presented differences with the PH and the TAA groups (F2, 25 = 16.954, *P* < 0.001). Finally, no differences were found in any of the mammillary nuclei explored: mMM (F2, 24 = 0.109, *P* = 0.898), lMM (F2, 24 = 1.368, *P* = 0.274), and LM (F2, 23 = 0.178, *P* = 0.838). See Table 1.

## 4. Discussion

The rationale for the present study arose from evidence that cerebral metabolic rate for glucose has consistently been reported to be decreased in patients with chronic HE, suggesting that hypometabolism contributes to the neuropsychiatric symptoms commonly observed in HE. In fact, it has been shown that cerebral oxygen metabolism and blood flow were decreased in cirrhotic patients with HE [28], and cognitive deficits are closely related to aberrant baseline brain activity measured by fMRI in these patients [29]. However, the existence of a differential contribution of factors, such as portal hypertension, hyperammonemia, and liver disease, to brain metabolic activity has not yet been explored. In this study, we demonstrated that each of these factors affects brain energy requirements to a different extent.

Several studies have assessed these experimental models in a spatial reference memory task. Whereas the portal hypertension model showed only mild impairment in this learning process [23], hyperammonemia and liver failure models were severely affected (Arias et al., unpublished results [30]).

Traditionally, the hippocampus has been widely shown to be involved in reference memory [31]. Specifically, it is important in establishing allocentric relations [32] and also seems to be responsible for correct goal recognition [33]. Together with this brain structure, prefrontal cortex, ventral striatum, anterior thalamus, and mammillary bodies cooperate in diverse memory processes and learning tasks [34, 35]. The role of the prefrontal cortex in the organization of spatially directed behaviours has been shown both in primates and humans. In rats, the main evidence comes from lesion studies in performance of various spatial tasks such as spatial alternation, spatial reversal, elimination in the radial maze, and navigation in the Morris water maze [36]. The connections between this prefrontal cortex and ventral striatum play a role in the transformation of route planning into motor response in memory tasks [37]. Likewise, the prefrontal cortex receives projections from the anterior thalamic nucleus [38], damage to which impairs allocentric memory tasks that are also disrupted by hippocampal dysfunctions [39]. The mammillary nuclei, which can be subdivided into the medial and lateral parts, are connected with the hippocampus and send projections to the anterior thalamus [40] and are involved in spatial memory processes [41, 42]. 

When we wondered about the possible time-dependent involvement of these structures in the spatial reference memory, we found that most of them were differentially implicated at the beginning and the conclusion of the task in nonpathological conditions [43]. That is, some structures, such as the anterior thalamic nuclei and the mammillary bodies, seem to be more involved in early task acquisition, but it is necessary for these structures to reduce their energy requirements in order to successfully complete the task. But other structures, such as DG, CA3, and the prefrontal cortex, showed an opposite pattern [43]. 

In view of this tendency, we were willing to explore the metabolic activity of the main contributing factors of HE. Accordingly to our expectations, the portal hypertension group reduced its metabolic activity in almost all studied regions compared to the other groups. A possible explanation for this behaviour, so similar to that of nonpathological subjects, is that, in the PH model, portosystemic collaterals develop as a consequence of high pressure in the portal vein and ameliorate the increased resistance [1, 44]. However, a different situation takes place in the HA group, in which the energetic demands increase above conventional levels. This increase in oxygen utilization could be due to an inflammation in these areas of the central nervous system [45]. Inflammation will increase the use of oxygen, represented by excess production of reactive oxygen and nitrogen species, which cause oxidative and nitrosative stress [46–48].

Several studies have shown that once ammonia crosses the blood brain barrier, it is immediately used to amidate glutamate to glutamine in the astrocytes in order to prevent its toxicity from damaging the neurons. But this depletion of the substrate of the tricarboxylic acid cycle together with the inhibition by the ammonia to rate limit enzymes such as pyruvate dehydrogenase and *α*-ketoglutarate will slow down overall oxidative metabolism, leading to depletion of energy-rich phosphate compounds [49]. Moreover, glutamatergic neurotransmission suffers disturbances owing to the fact that ammonia impairs the induction of NMDA receptors, which alters the neural glutamate-nitric oxide cyclic GMP pathway, in turn, involved in learning and memory [20, 21].

At the same time, glutamine, which is an osmotically powerful substance, increases due to ammonia in the brain and can provoke a rapid brain edema, triggering intracranial hypertension. During liver failure, high arterial ammonia levels lead to accumulation in the brain [50] and exert numerous deleterious effects, contributing to the clinical presentation of HE. Subsequent studies carried out in various animal models of acute and chronic HE, such as our TAA model, have reported altered glucose utilization similar to the HA models [51]. But, whereas patients with liver cirrhosis have a compensatory mechanism that prevents the occurrence of brain edema and intracranial hypertension [18], the hyperammonemic brain cannot establish these mechanisms.

Another possible mechanism for impaired energy metabolism in HA is the mitochondrial permeability transition of the inner membrane, which has a critical linkage with disturbed cerebral energy metabolism and oxidative/nitrosative stress [51]. Similar to free radicals, nitric oxide increases in the brain of the TAA-administration rat model [52]. A possible explanation for the alteration of COx activity would be the increased level of oxidative stress [45]. Liver disease is associated with the production of free radicals that override the balance between oxidative stress and antioxidative mechanisms, leading to oxidative stress. Oxidative stress could play a role in the pathogenesis of HE, due to the fact that the brain of patients with HE is particularly susceptible to oxidative damage [47]. Peroxidase damage and increased oxidative stress have been reported in neural membranes of rats with thioacetamide-induced HE [53] and in diverse central nervous system regions of the same animal model [54]. Brain oxidative and nitrosative (superoxide anion with nitric oxide produces peroxynitrite) stress in cirrhotic rats could cause neural mitochondrial damage and defective oxidative phosphorylation, which is reflected in impaired COx activity. Therefore, oxidative and nitrosative stress caused activation of the nuclear factor KB, which is the major inducer of proinflammatory cytokines and chemokines.

The differences found in bioenergetic requirements could indicate a differential affectation of the neural networks underlying different behaviours, so a broad field of future research opens up.

In conclusion, the results obtained show that there is a differential contribution of portal hypertension, hyperammonemia, and liver disease to the brain metabolic dysfunction associated with HE. The most interesting finding is that the alterations in metabolic brain activity do not develop equally in the three models. The severest alteration is found in the model that mimics intoxication by ammonia, where the main cerebral structures have to make a big effort in comparison with the other two experimental models. 

## Figures and Tables

**Table 1:** Metabolic activity of the selected brain regions in the studied groups.

	PH	HA	TAA
*Prefrontal cortex *			
PL	18.364 ± 0.460^a,b^	26.781 ± 1.032	22.591 ± 0.379^a^
IL	18.470 ± 0.504^a^	26.988 ± 0.916	19.432 ± 0.401^a^
*Ventral Striatum *			
AcC	21.362 ± 0.395^a,b^	31.812 ± 1.032	25.552 ± 0.498^a^
AcS	22.210 ± 0.748^a,b^	36.940 ± 1.227	32.409 ± 0.715^a^
*Thalamic nuclei *			
ADT	30.525 ± 0.736^a,b^	50.930 ± 2.024	40.025 ± 1.302^a^
AVT	24.717 ± 0.525^a^	31.753 ± 1.412	27.631 ± 1.120^a^
MDT	19.307 ± 0.879^a^	27.361 ± 0.809	18.242 ± 0.921^a^
*Hippocampus *			
DG	23.413 ± 0.500^a,b^	39.550 ± 1.75	32.663 ± 1.739^a^
CA3	16.318 ± 0.488^a,b^	22.633 ± 0.763	12.592 ± 0.568^a^
CA1	18.128 ± 0.707^a^	24.820 ± 0.915	18.250 ± 0.915^a^
*Mammillary nuclei *			
mMM	22.949 ± 1.151	23.490 ± 1.336	23.769 ± 1.479
lMM	17.643 ± 0.950	16.792 ± 0.633	15.777 ± 0.597
LM	25.923 ± 1.113	26.624 ± 1.246	26.665 ± 0.489

Values are mean ± SEM.

PH: portal hypertension, HA: hyperammonemia, TAA: thioacetamide.

^a^ *P* < 0.05 statistically significant value in relation to HA.

^b^ *P* < 0.05 statistically significant value in relation to TAA.
